# The regulatory role of placental extracellular vesicle on trophoblast and endothelial cell functions

**DOI:** 10.3389/fcell.2025.1528714

**Published:** 2025-02-10

**Authors:** Kunfeng Bai, Xintong Li, Yanjie Guo, Ye Shang, Leqian Lin, Philip C. N. Chiu, Cheuk-Lun Lee

**Affiliations:** ^1^ Guangzhou Women and Children’s Medical Center, Institute of Pediatrics, Guangzhou, China; ^2^ Department of Obstetrics and Gynaecology, The University of Hong Kong, Pokfulam, Hong Kong SAR, China; ^3^ Shenzhen Key Laboratory of Fertility Regulation, The University of Hong Kong – Shenzhen Hospital, Shenzhen, China; ^4^ Department of Health Technology and Informatics, The Hong Kong Polytechnic University, Kowloon, Hong Kong SAR, China

**Keywords:** extracellular vesicle, trophoblast, endothelial cells, spiral artery remodeling, CD147, pregnancy, placenta, siglec-6

## Abstract

Extracellular vesicles (EVs) are cell-derived, membrane-bound vesicles that carry molecular cargo to facilitate communication between cells. During pregnancy, EVs are secreted by the syncytiotrophoblast layer of the placenta villi, where they mediate the functions of resident leukocytes and invading extravillous trophoblasts (EVTs) in the decidua. This study aims to isolate placental EVs (pEVs) from placental explant to examine their regulatory roles on EVT and endothelial cell functions. pEVs were successfully isolated from *ex vivo* cultured placental explant, which were capable to be internalized by EVTs and endothelial cells. pEVs stimulated the differentiation of trophoblast stem cells (TSCs) and enhanced the migration and invasion abilities of EVTs via CD147 receptor. Conversely, pEVs inhibited the tube formation ability and interleukin-6 (IL-6) secretion of endothelial cells. Together, these findings partially elucidate the role of pEVs during early pregnancy establishment, which may provide insights into pregnancy-related disorders.

## 1 Introduction

During early pregnancy, extensive crosstalk between the maternal decidua and the fetal placenta is crucial for successful establishment of maternal-fetal interface and ensuring the progression of pregnancy (Li et al., 2022). Following embryo implantation, the trophectoderm of the blastocyst begins to form the placenta, which serves as the source of nutrition and facilitates maternal-fetal exchange throughout the pregnancy. The basic units of the placental villi, called cytotrophoblasts (CTBs), are covered by a layer of multi-nucleated syncytiotrophoblasts (STBs), which are primarily responsible for hormone secretion. CTBs also differentiate into extravillous trophoblasts (EVTs) that migrate and invade the maternal decidua and spiral arteries, allowing the anchoring of placenta and remodelling of spiral arteries followed by regulation of endothelial functions, which together contribute to successful pregnancy establishment (Cindrova-Davies and Sferruzzi-Perri, 2022). Throughout this process, various intercellular communication pathways are involved at the maternal-fetal interface, including those mediated by extracellular vesicles (EVs).

EVs are membrane-bound vesicles derived from cells that carry molecular cargos, such as miRNAs and proteins, to facilitate communication between cells (Sheta et al., 2023). Throughout gestation, they are mainly secreted by STBs in the placenta into the maternal bloodstream. These EVs play a crucial role in regulating maternal immune tolerance and placental development, particularly affecting the functions of EVTs and endothelial cells (Paul et al., 2023). Although some studies have highlighted the regulatory roles of EVs in early pregnancy, their specific effects on trophoblast differentiation, migration, invasion, and endothelial cell functions during placental development are not yet fully understood.

CD147 is a key component of trophoblast membrane complexes and plays a regulatory role in the differentiation and spiral artery remodeling of EVTs (Lee et al., 2022). Sialic acid-binding Ig-like lectin-6 (siglec-6) is a glycan receptor specifically expressed in the human placenta (Zhong et al., 2024), with its expression levels increasing during labor (Brinkman-Van der Linden et al., 2007). Both CD147 and siglec-6 are involved in cell recognition, differentiation, invasion, and angiogenesis. Increased expression of siglec-6 in placental extracellular vesicles (pEVs) (Li et al., 2015) and decreased levels of soluble CD147 (Lee et al., 2022) have been linked to preeclampsia, a serious pregnancy complication that is a leading cause of maternal and perinatal morbidity and mortality, as well as long-term health complications.

This study aims to elucidate the regulatory roles of pEVs, along with CD147 and siglec-6 on pEVs, in the functions of EVTs and endothelial cells during the establishment of early pregnancy.

## 2 Materials and methods

### 2.1 Isolation of pEV

Human placenta samples were collected from women undergoing surgical abortion at 7th to 10th weeks with written consent. This study was approved by the Institutional Review Board (IRB) of The University of Hong Kong/Hospital Authority Hong Kong West Cluster (IRB no.: UW 22-013). Placental explant was cultured according to previously described methods (Bai et al., 2022; Bai et al., 2023). Briefly, placenta villi were isolated and cultured in 40 μm cell strainer at 37°C, 2% oxygen environment. The culture medium was replaced 3 h after culture to remove cell debris and apoptotic bodies. The villi explant was cultured for 40 h for conditioned medium collection. The conditioned medium was first centrifuged at 300 g for 10 min, followed by 2,000 g for 20 min to remove cell debris. The supernatant was then centrifuged at 120,000 g for 120 min at 4°C for pEV isolation and washed in PBS twice. Protein concentrations of the isolated pEVs were determined by bicinchoninic acid (BCA) assay. Characterization of pEVs was conducted by Western blot analysis, electron microscopy and nanoparticle tracking analysis (NTA) according to previously published methods (Bai et al., 2022; Bai et al., 2023).

### 2.2 Endothelial cell culture

Human umbilical vein endothelial cells (HUVECs) were obtained from Lonza (Lonza, C2517A) and cultured in F12K (Sigma Aldrich) supplemented with 10% FBS, 30 μg/mL of endothelial cell growth supplements and 0.1 mg/mL of heparin at 37°C, 5% CO_2_ environment.

### 2.3 Human trophoblast stem cell (TSC) culture

Human TSC (hTSC) line was established from human expanded potential stem cells (hEPSCs) according to previously described method, namely, TSC-like cells (Zhang et al., 2023). TSC-like cells were cultured in 1% Geltrex-coated plate at 37°C in TSC medium (Dulbecco’s modified Eagle’s medium (DMEM)/F12 supplemented with 50 μM β-mercaptoethanol, 0.2% FBS, 0.5% penicillin-streptomycin-glutamine (PSG), 0.3% Bovine serum albumin (BSA), 1% Insulin-Transferrin-Selenium-Ethanolamine (ITS-X) supplement, 50 μg/ml L-ascorbic acid, 50 ng/mL epidermal growth factor (EGF), 2 μM CHIR99021, 0.5 μM A83-01, 1 μM SB431542, 10 μM Valproic acid (VPA), and 5 μM Y27632). TSC-like cells were subcultured every 3–4 days with 1 mL of TrypLE.

### 2.4 TSC-like cells differentiation

After TSC-like cells reached 80% confluency, they were digested with TrypLE for 5 min at 37°C for induction of differentiation into EVTs (TSC-EVTs). TSC-like cells were seeded at a density of 1 × 10^5^ cells per well in 1% Matrigel-coated 6-well plate in 2 mL of EVT medium (DMEM/F12 supplemented with 50 μM β-mercaptoethanol, PSG, 0.3% BSA, 1% ITS- X, 7.5 μM A83-01, 10 μM Y27632, 4% KnockOut Serum Replacement (KSR), 100 ng/mL neuregulin 1 (NRG1) and 2% Matrigel). The differentiation medium was changed on day 3 (DMEM/F12 supplemented with 50 μM β-mercaptoethanol, PSG, 0.3% BSA, 1% ITS- X, 7.5 μM A83-01, 10 μM Y27632, 4% KSR and 0.5% Matrigel). TSC-EVTs were harvested on day 6 for downstream analysis.

### 2.5 pEV internalization by TSC-EVTs and HUVECs

For pEV internalization by TSC-EVTs, pEVs were labeled with PKH26-Red Fluorescent cell linker kit (Sigma-Aldrich) according to the manufacturer’s protocol. In brief, pEVs were resuspended with Diluent C solution after washing. Protein concentration was determined with BCA assay. pEVs were stained with fluorescent dye for 5 min and washed by PBS. TSC-EVTs were incubated with 10 μg/mL of pEVs diluted in the complete medium for 4 h, followed by washing with PBS and fixed with 4% PFA. The negative control group was treated with Diluent C at the same volume. TSC-EVTs were permeabilized with 1x Permeabilizing buffer, blocked with 10% goat serum and incubated with primary antibody against CK7 (1:200, CBL194F, Sigma) overnight at 4°C, followed by Goat anti-Mouse IgG (H+L) Superclonal™ Recombinant Secondary Antibody, Alexa Fluor^®^ 488 conjugate (1:600, A28175, Invitrogen) for 1 h at room temperature. The cells were counterstained with DAPI for 5 min and visualized under fluorescence microscopy.

For pEV internalization by HUVECs, pEVs were labeled with PKH67-Green Fluorescent cell linker kit (Sigma-Aldrich) according to the protocol mentioned above. HUVECs were stained with CellTrackerRed (Thermo Scientific) by incubation in 500 μL of working solution (2.5 μL CellTrackerRed diluted in 2.5 mL blank medium) for 30 min at 37°C. HUVECs were treated with 10 μg/mL of pEVs diluted in the complete culture medium for 4 h, while the negative control group was treated with Diluent C at the same volume. After 4 h of incubation, the culture medium was removed and HUVECs were rinsed with cold PBS three times. Cells were fixed with 4% PFA, counterstained with DAPI for 5 min and visualized under the fluorescence microscope.

### 2.6 Tube formation assay

HUVECs were treated with 10 μg/mL of pEVs for 24 h. After that, pEV-treated HUVECs were seeded at a density of 10,000 cells on μ-slide angiogenesis chamber with Matrigel coating (Corning) and cultured for 6 h for tube formation. Images were captured under inverted microscope and tube formation was analyzed using Image-Pro-Plus software (Media cybernetics) with the angiogenesis plugin.

### 2.7 Human cytokine antibody array

Conditioned medium of pEV-treated HUVECs was collected after 24 h for cytokine array using a human cytokine antibody array kit (ARY005B, R&D) according to manufacturer’s instructions. Briefly, the membrane was blocked with 2 mL of blocking reagent for 1 h, and 1 mL of diluted conditioned medium was added to the membranes and incubated at 4°C overnight. The membrane was washed in wash buffers and incubated in biotin-conjugated detection antibody for 2 h, followed by incubation in HRP-conjugated streptavidin for 30 min. The spots were developed with Chemi Reagent Mix using X-ray films, and the intensity of each cytokine-representing spot was measured by ImageJ software with normalization to positive control spots.

### 2.8 Enzyme-linked immunosorbent assay (ELISA)

The concentration of IL-6 in conditioned medium of pEV-treated HUVECs was analyzed using ELISA kit (eBioscience) under manufacturer’s instruction. In brief, 96-well culture plate was coated with primary antibody against IL-6 and incubated at 4°C overnight, then blocked with assay buffer for 2 h after washing. One-hundred microliters of conditioned medium was added to the plates with 50 μL of detection antibody and incubated for 16 h at 4°C, followed by incubation with HRP-conjugated streptavidin for 30 min after washing. The plates were then incubated with 100 μL of 3, 3′, 5, 5′-tetramethylbenzidine (TMB, BD Biosciences) substrate for 15 min, stopped by 50 μL of 1.8 N sulfuric acid, and read at 450 nm with a reference wavelength of 595 nm.

### 2.9 Flow cytometry

To conjugate pEVs with antibodies, pEVs were first incubated with anti-placental alkaline phosphatase (PLAP, ab133602, Abcam)-conjugated magnetic beads (20 μL/mL, Dynabeads™, Invitrogen) for 2 h to ensure their placental origin. AF647-labelled antibodies, including isotype antibody, siglec-6 antibody (ab262851, Abcam) and CD147 antibody (ab666, Abcam), were incubated with anti-PLAP bead-linked pEVs (10 μg) for 2 h. Antibody-conjugated pEVs were examined by flow cytometry to confirm the successful binding of siglec-6 and CD147 antibody. Fluorescence signal of labelled pEVs was detected with CytoFLEX flow cytometer (Beckman Coulter). Data was analyzed with FlowJo software (Tree Star).

### 2.10 Real-time quantitative polymerase chain reaction (RT-qPCR)

TSC-like cells were treated with pEVs in the presence of isotype, siglec-6 and CD147 antibodies at 10 μg/mL on day 0 and day 3 during differentiation into TSC-EVTs. Total RNA of TSC-like cells, TSC-EVTs and TSC-EVTs treated with pEVs was extracted by the QuickPrep RNA extraction kit (GE Healthcare) and reversely transcribed by the TaqMan Reverse Transcription Reagent (Takara). qPCR was conducted with QuantStudio 5 Real-Time PCR System (Applied Biosystems) using TaqMan qPCR assay probes including EVT markers Human leukocyte antigen G (*HLAG*, Hs00365950_g1), Matrix metallopeptidase 2 (*MMP2*, Hs01548727_m1) and Integrin alpha 5 (*ITGA5*, Hs01547673_m1), pan-trophoblast marker Cytokeratin 7 (*KRT7*, Hs00559840_m1), and 18S ribosomal RNA (Hs99999901_s1) as internal control. Calculation of relative expression level was conducted with the threshold cycle (CT) method (2^−△△CT^ method).

### 2.11 Transwell invasion and migration assay

TSC-EVTs were collected for transwell invasion assay using Matrigel Invasion Chamber 24-well plate 8.0 Micron (Corning), and for transwell migration assay using 8 μm culture insert (Merck) and 24-well culture plate. TSC-EVTs were seeded at a density of 10,000 cells in 300 μL of blank medium supplemented with 10 μg/mL of pEVs in the presence of isotype, siglec-6 and CD147 antibodies in the upper chamber for invasion assay, and at a density of 8,000 cells for migration assay. A control group was set with identical cell number but supplemented with blank medium in the upper chamber. The lower chamber was filled with 10% FBS diluted in blank medium. The invasion and migration system were cultured at 37°C for 20 h separately. The chambers were stained in 0.5% Crystal Violet for 15 min, followed by washing and removal of non-invaded cells using cotton sticks. Images of the lower chamber were captured, and the mean number of invaded/migrated cells was analyzed using ImageJ software (US National Institute of Health). The relative invasion/migration ratio was calculated by dividing the invasion/migration ratio of control group without pEV treatment.

### 2.12 Statistical analysis

All the data was presented as means ± standard deviation. All the data were analyzed by the Kolmogrov–Smirnov normality test followed by unpaired Student’s t-test or one-way analysis of variance (ANOVA) with multiple comparison using Graph Pad Prism 9 software (Graph Pad Software Inc.). P < 0.05 was considered as a statistically significant difference.

## 3 Results

### 3.1 pEVs were successfully isolated from placental explant and internalized by TSC-EVTs and HUVECs

**FIGURE 1:**
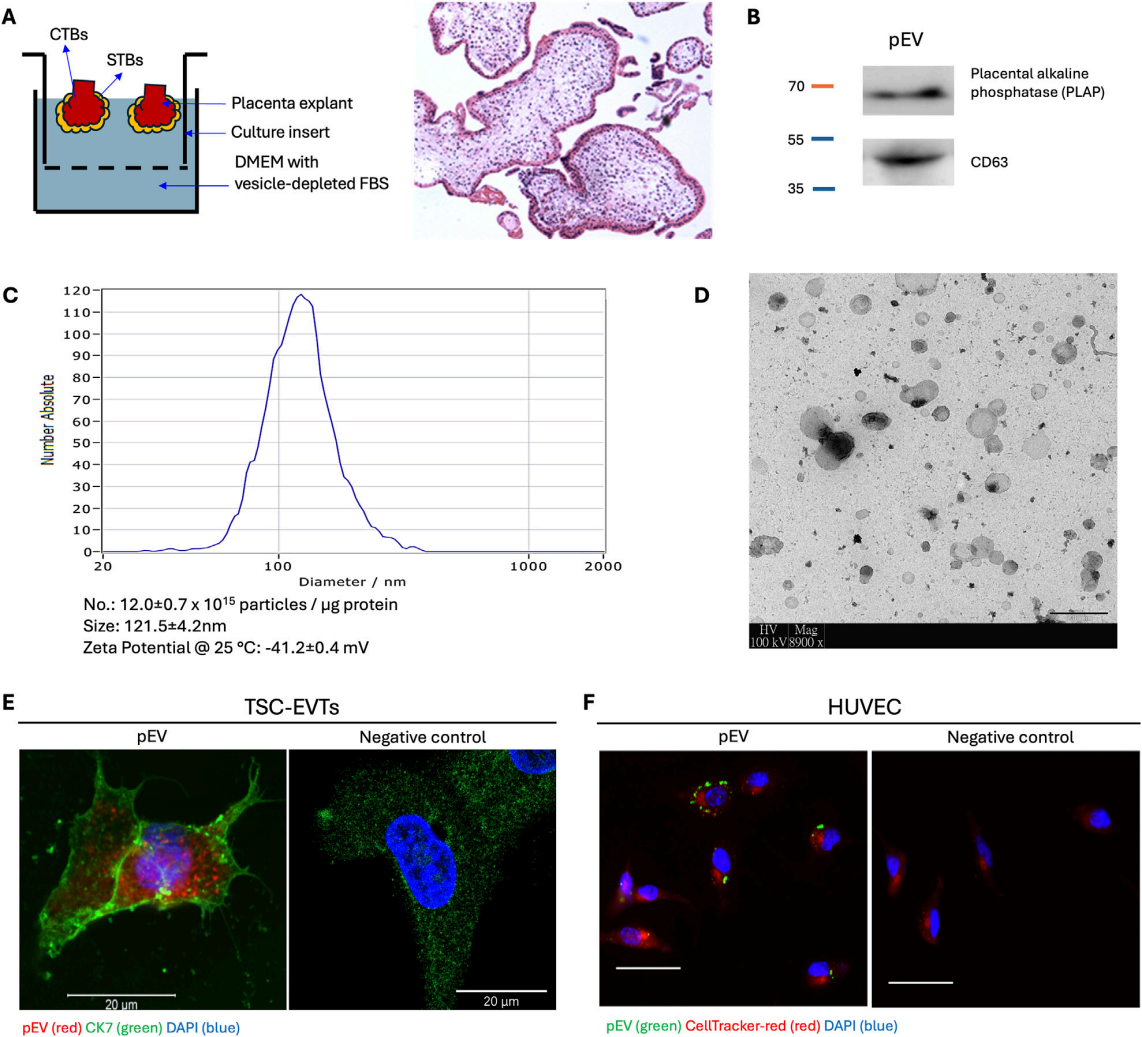
Isolation, characterization and internalization of placental extracellular vesicles (pEVs). **(A)** Schematic illustration of *ex vivo* culture of placenta villi under hypoxia (2% O_2_) condition. Hematoxylin and Eosin (H&E) staining of placenta villi after *ex vivo* culture. CTB: cytotrophoblast. STB: syncytiotrophoblast. **(B)** Western blot analysis for PLAP (placenta origin) and CD63 (EV marker) in isolated pEVs. **(C)** Nanoparticle tracking analysis (NTA) for the isolated pEVs. Particle concentration of pEVs is approximately 12.0 ± 0.7 × 1,015 particles/μg pEV protein. The mean size is 121.5 ± 4.2 nm with a Zeta potential of −41.2 ± 0.4 mV. **(D)** Transmission electronic microscope image for isolated pEVs. Scale bar = 500 nm. **(E)** Fluorescence microscopy image of TSC-EVTs treated with fluorescence PKH26-labelled pEVs demonstrating internalization of pEVs. Negative control was incubated with blank Diluent C in PKH26 kit. Red: pEV; Green: Cytokeratin 7 (CK7); Blue: DAPI. Scale bar = 20 μm. **(F)** Fluorescence microscopy images of HUVECs treated with PKH67-labelled pEVs demonstrating internalization of pEVs by HUVECs. Negative control was incubated with blank Diluent C in PKH67 kit. Red: HUVECs; Green: pEVs; Blue: DAPI. Scale bars = 100 μm.

To isolate pEVs, an *ex vivo* culture system of placental explant was established (Bai et al., 2022; Bai et al., 2023) (Figure 1A). The isolated pEVs showed the positive expression of placental marker PLAP and EV marker CD63 (Figure 1B). Furthermore, NTA and electron microscopy result confirmed the size and morphology of pEVs, which showed majority of the particles fell into the size range from 100 to 500 nm (Figures 1C, D), together demonstrating the successful isolation of pEVs.

To study the ability of pEV uptake, TSC-EVTs and HUVECs were incubated with pEVs supplemented in the culture medium for 4 h followed by fluorescence signal capture. TSC-EVTs showed prominent fluorescence signals of pEVs labelled with PKH26, and the positive expression of CK7 demonstrated their trophoblast origin, while the negative control group incubated with blank medium did not show fluorescence signal of pEVs (Figure 1E). Likewise, HUVECs showed positive fluorescence signals for PKH67-labelled pEVs, while the negative control group only showed the red signal for CellTracker-red (Figure 1F). Together, pEVs were capable to be internalized by TSC-EVTs and HUVECs from the culture medium.

### 3.2 pEVs inhibited tube formation and IL-6 secretion in endothelial cells

**FIGURE 2:**
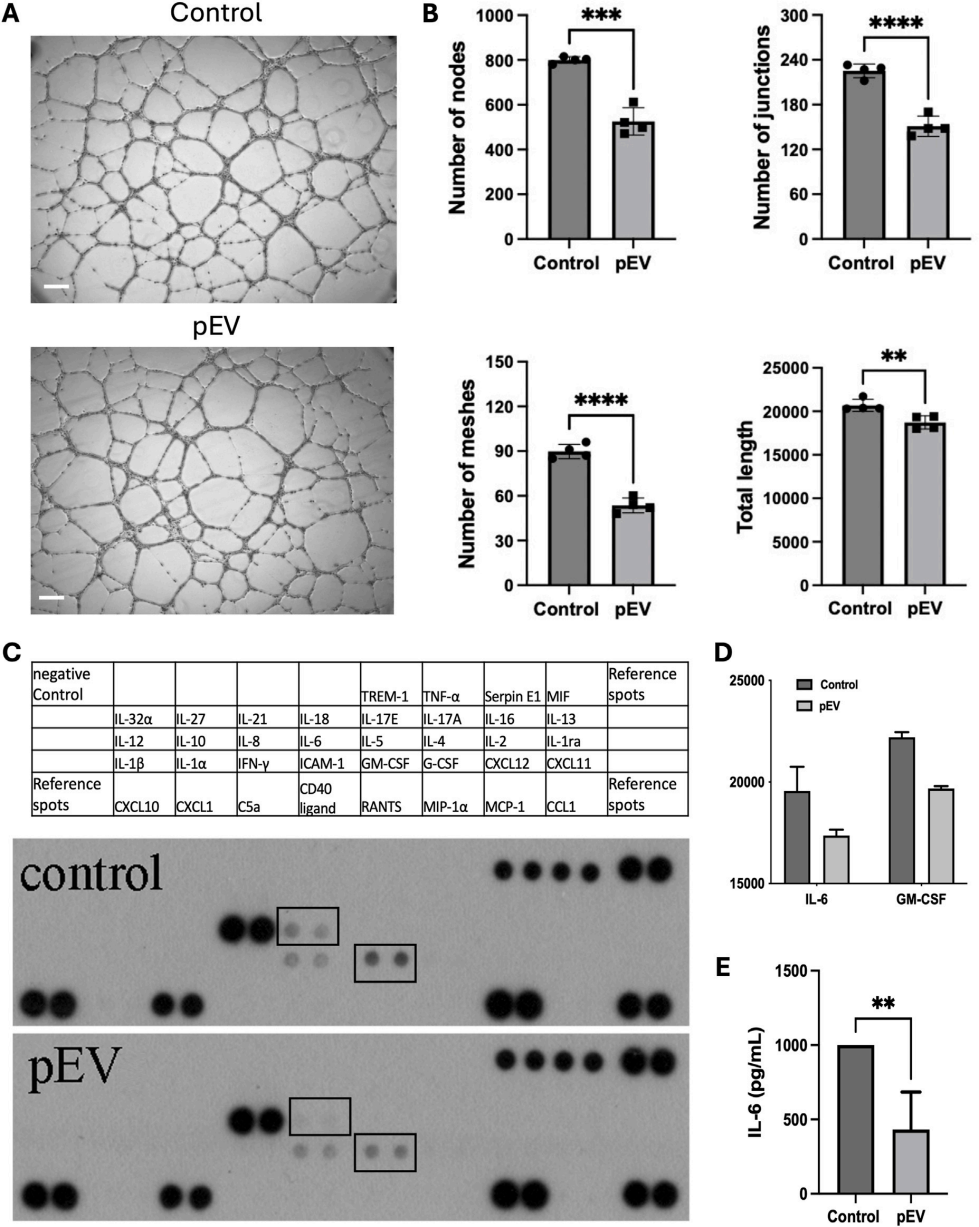
pEVs inhibited endothelial cell activation *in vitro*, with suppressed secretion of IL-6 in pEV-treated HUVECs. **(A)** Representative images for tube formation assay of HUVECs treated with 10 μg/mL of pEVs for 24 h. Scale bars = 100 μm. **(B)** Number of nodes, number of junctions, number of meshes, total length in endothelial network formed by HUVECs treated with 10 μg/mL of pEVs for 24 h. N = 4. **: P < 0.01, ***: P < 0.001, ****: P < 0.0001. **(C)** Cytokine array for conditioned medium collected from HUVECs treated with 10 μg/mL of pEVs for 24 h. **(D)** Intensity of IL-6 and GM-CSF in cytokine array for conditioned medium collected from pEV-treated HUVECs. N = 2. **(E)** ELISA analysis for the expression level of IL-6 in conditioned medium collected from pEV-treated HUVECs. N = 4. **: P < 0.01.

To understand the role of pEVs on endothelial cell functions, HUVECs were treated with 10 μg/mL of pEVs for 24 h. The pEVs treatment significantly inhibited the tube formation ability of HUVECs, with a reduced number of nodes, junctions, meshes, and total length of tubular structures compared to the control group (Figures 2A, B).

To further assess the effect of pEVs on cytokine production by HUVECs, the conditioned medium from pEV-treated HUVECs showed lower levels of IL-6 and Granulocyte-Macrophage Colony-Stimulating Factor (GM-CSF) compared to the ones from the control group (Figures 2C, D). This reduced expression of IL-6 was further confirmed by ELISA (Figure 2E). These findings suggested that pEVs inhibit endothelial cell tube formation and IL-6 production, potentially playing a role in maintaining homeostasis during the early stages for endothelial function regulation.

### 3.3 pEVs stimulated TSC differentiation, EVT invasion and migration via CD147

**FIGURE 3:**
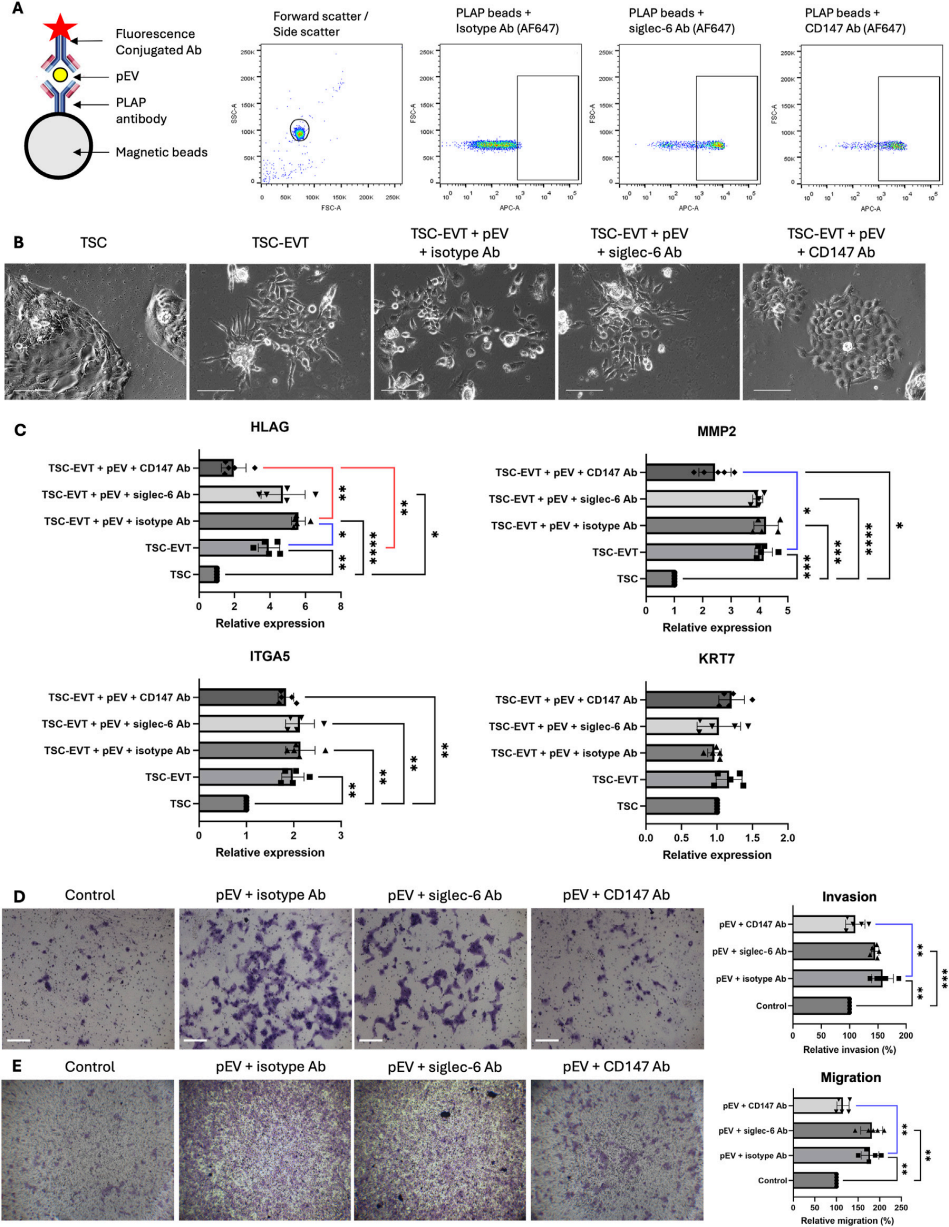
pEVs stimulated EVT differentiation, invasion and migration via CD147 expression *in vitro*. **(A)** Schematic illustration for pEV antibody conjugation. Anti-PLAP-conjugated beads were incubated with pEV and subsequently with fluorescence (AF647)-conjugated isotype antibody, siglec-6 antibody and CD147 antibody for flow cytometry analysis, demonstrating the expression of Siglec-6 and CD147 on pEV. Ab: antibody. **(B)** Representative images of TSC-like cells, EVT differentiated from TSC (TSC-EVT) and TSC-EVTs treated with pEVs in the presence of isotype antibody, siglec-6 antibody and CD147 antibody. Scale bar = 100 μm. **(C)** Relative mRNA expression levels of EVT markers HLAG, MMP2, ITGA5 and pan trophoblast marker KRT7 in TSC, TSC-EVT and TSC-EVTs treated with 10 μg/mL of pEVs in the presence of isotype antibody, siglec-6 antibody and CD147 antibody. N = 5. *: P < 0.05, **: P < 0.01, ***: P < 0.001, ****: P < 0.0001. **(D)** Invasion assay for EVTs treated with 10 μg/mL of pEVs in the presence of isotype antibody, siglec-6 antibody and CD147 antibody with representative images and relative invasion ratio. N = 5. **: P < 0.01, ***: P < 0.001. Scale bars = 100 μm. **(E)** Migration assay for EVTs treated with 10 μg/mL of pEVs in the presence of isotype antibody, siglec-6 antibody and CD147 antibody with representative images and relative migration ratio. N = 5. **: P < 0.01. Scale bars = 100 μm.

To investigate the presence of CD147 and siglec-6 on pEVs, we first conjugated pEVs with PLAP antibody-linked magnetic beads to confirm their placental origin. Subsequently, fluorescence-conjugated antibodies against CD147 and siglec-6 were attached, along with an isotype antibody as a negative control. Flow cytometry result confirmed the successful loading of antibodies with the PLAP bead (Figure 3A).

To investigate the impact of pEVs on TSC differentiation, TSC-like cells were treated with pEVs conjugated with antibodies against siglec-6/CD147 or isotypic antibodies during their differentiation into EVTs (TSC-EVTs). Treatment with pEV at a concentration of 10 μg/mL promoted the differentiation of TSC-like cells into TSCs-EVTs, with an increased expression of HLAG when compared to TSC-like cells without pEV treatment. This stimulatory effect was abolished when CD147 in pEVs was blocked by antibodies, but not by antibodies against siglec-6. There were no significant differences in the expression levels of MMP2 and ITGA5 between pEV-treated TSC-EVTs and TSC-EVTs, nor in the expression of the pan-trophoblast marker KRT7 (Figures 3B, C). Furthermore, to study the invasion and migration ability of TSC-EVTs upon pEV treatment, treating TSC-EVTs with 10 μg/mL of pEVs significantly enhanced their cell migration and invasiveness compared to the control group incubated with identical volume of blank medium. However, this effect was abolished when CD147 was blocked in the pEVs (Figures 3D, E). In summary, pEVs promote the differentiation, migration, and invasion of TSC-EVTs *in vitro*, likely through the action of CD147 on the pEVs.

## 4 Discussion

Early placentation is a critical process in establishing a successful pregnancy, involving intricate interactions between trophoblasts and endothelial cells during the spiral artery remodeling process. pEVs derived from the syncytiotrophoblast layer of the placenta villi play a pivotal role in mediating this process. In this study, pEVs were successfully isolated from an *ex vivo* placental explant model, which could be internalized by *in vitro* cultured EVTs and endothelial cells. In endothelial cells, pEVs inhibited the tube formation ability and production of IL-6 in HUVECs. In trophoblast cells, pEVs enhanced the differentiation of TSC-like cells into EVTs, as well as the migration and invasion of EVTs. This effect was abrogated when CD147 on pEVs was blocked. Collectively, these findings partially revealed the regulatory role of pEVs in early placentation and the establishment of the maternal-fetal interface.

To isolate the EVs derived from placenta, an *ex vivo* culture system of placental explants was established (Bai et al., 2021). Currently, explant culture of first trimester placenta is a valuable model for studying early placentation. Oxygen tension has been demonstrated to be an essential factor for trophoblast proliferation, differentiation and syncytiolization. In this study, all the placenta samples were collected from surgical termination of pregnancy at first trimester (7–10 weeks) to minimize variability and mimic the period of early placentation. The placenta explant was cultured under 2% O_2_ since oxygen pressure at the early maternal-fetal interface is about 2% (Jauniaux et al., 2000). Another study also demonstrated that the placenta under a hypoxic environment releases much more exosomes than the normoxic environment (Salomon et al., 2013).

A key step to a successful pregnancy is the transformation of high-resistance muscular maternal arteries into low-resistance, high-flow vessels capable of providing sufficient placental perfusion to support the growing fetus throughout pregnancy (Su and Fazleabas, 2015). This process, termed as remodeling of the spiral arteries, occurs during the first 12 weeks of pregnancy. It involves the differentiation and subsequent invasion of EVTs into the spiral arteries, leading to the replacement of the arterial lining. Failure of this remodeling process can lead to pregnancy-associated diseases such as preeclampsia and fetal growth restriction (Rana et al., 2019).

In this study, pEVs enhanced the differentiation of TSCs into EVTs, as well as the migration and invasion abilities of TSC-EVTs. This underscores the significant role of pEVs in mediating cell-to-cell communication during early placentation. Placental villi release EVs that can act in an autocrine or paracrine manner to influence EVT differentiation and function in the maternal decidua, promoting vascular remodeling and modulating metabolic adaptations during pregnancy (Ouyang et al., 2014; Tannetta et al., 2017). The enhanced differentiation of TSCs into EVTs upon pEV treatment suggests that pEVs may deliver specific signals or cargo molecules, such as proteins and microRNAs (miRNAs), that facilitate trophoblast lineage commitment and functional maturation, including invasion and migration (Luo et al., 2009). Our previous study has identified the major miRNA content in pEVs isolated from the same source and procedure, for example, miR-143-3p, miR-148a-3p and miR-24-3p (Bai et al., 2023). These miRNAs are reported to regulate cellular homeostasis, cell differentiation and endothelial functions (Zhang et al., 2020; Kim et al., 2019; Wu et al., 2024), which may help to explain the role of pEVs for placental development regulation.

The role of CD147 in this process is particularly noteworthy. The presence of CD147 on the surface of pEVs suggests that they may interact directly with CD147-binding partners on trophoblasts, triggering downstream signaling cascades that promote differentiation and invasion. In trophoblasts, CD147 has been implicated in promoting cell invasion and migration, partly by upregulating matrix metalloproteinase activity. Our previous study (Lee et al., 2022) demonstrated that CD147 regulates trophoblast functions via activation of the Wnt/β‐catenin signaling pathway. We showed that CD147 enhances EVT differentiation, migration, invasion and matrix metalloproteinase activities by activating Wnt/β‐catenin signaling. Therefore, the finding that blocking CD147 on pEVs abrogated the stimulatory effects on EVT differentiation and invasion in the present study may be explained by the disruption of CD147-mediated activation of Wnt/β‐catenin signaling in trophoblasts. This indicates that CD147 on pEVs is a key mediator of trophoblast invasive properties and plays a significant role in spiral artery remodeling during early pregnancy.

In contrast to the stimulatory effects on EVTs, pEVs inhibited endothelial cell functions, as evidenced by reduced tube formation ability and decreased production of IL-6 in HUVECs. This aligns with the concept that pEVs exert differential effects on various cell types at the maternal-fetal interface, potentially contributing to the fine-tuning of placental vascular development. Conversely, a study conducted by Michelle O’brien et al., revealed that soluble factors but not EVs influence endothelial cell functions (O'Brien et al., 2017). The difference may come from the different EV preparation methods and experimental conditions. To mimic the microenvironment of maternal-fetal interface during early pregnancy, placenta explants were cultured under a hypoxic chamber for 40 h (2% O_2_). While in O'Brien et al., placenta explants were cultured for 72 h under 8% O_2_. Of note, the oxygen concentration has a critical impact on trophoblast functions (Mouillet et al., 2010; Chen et al., 2006; Nelson et al., 1999).

Endothelial cells have been shown to produce multiple cytokines and chemokines in various physiological processes. The crosstalk between the placenta and the endothelium may be critical to the early placenta and fetal development (Sen et al., 2013). In this study, IL-6 secretion was suppressed by pEVs. A previous study suggested that pEVs from patients with gestational diabetes mellitus (GDM) activate endothelial cells, leading to high level of cytokine secretion, including IL-4, IL-6, IL-8, IFN-γ, TNF-α (Salomon et al., 2016). This discrepancy might come from the different sample sources and dosage, as pEVs in our study were isolated from the first-trimester placenta from patients who undergo termination of pregnancy procedures.

In our study, a few limitations are present. Firstly, pEVs used in the experiment were only derived from *ex vivo* cultured villi tissue. Other trophoblast models, for example, trophoblast organoids and human TSCs, may be used as EV sources to further confirm the consistency of the result. Secondly, as our result mainly involve the *in vitro* experiments, *in vivo* validations, for example, the mouse studies, may be attempted in the future. In our study, the potential of pEVs to be used as a treatment strategy for diseases related to trophoblast and endothelial dysfunction during early pregnancy has been revealed, which might be applied for clinical use in the future.

In summary, our study demonstrates that pEVs play important roles in modulating trophoblast and endothelial cell functions during early placentation. According to our findings, pEVs promote trophoblast differentiation and invasiveness, which is likely through CD147-mediated mechanisms, while inhibiting endothelial cell angiogenic activity and cytokine secretion. These findings provide new insights into the complex intercellular communication at the maternal-fetal interface mediated by EVs. Further elucidation of the molecular pathways involved may contribute to the development of detection and therapeutic strategies for pregnancy-related complications associated with abnormal placentation.

## Data Availability

The raw data supporting the conclusions of this article will be made available by the authors, without undue reservation.

## Supplementary material

SUPPLEMENTARY FIGURE 1 TSCs show high purity in terms of GATA3 expression. Immunofluorescence staining of GATA3 (green) in TSCs. The nuclei were counterstained with DAPI (blue). Scale bar = 20 μm.
