# Supplementary figures and images for: The regulatory role of placental extracellular vesicle on trophoblast and endothelial cell functions
Source: Front Cell Dev Biol. 2025 Feb 10;13:1528714. doi: 10.3389/fcell.2025.1528714 (PMC11847863; doi:10.3389/fcell.2025.1528714)

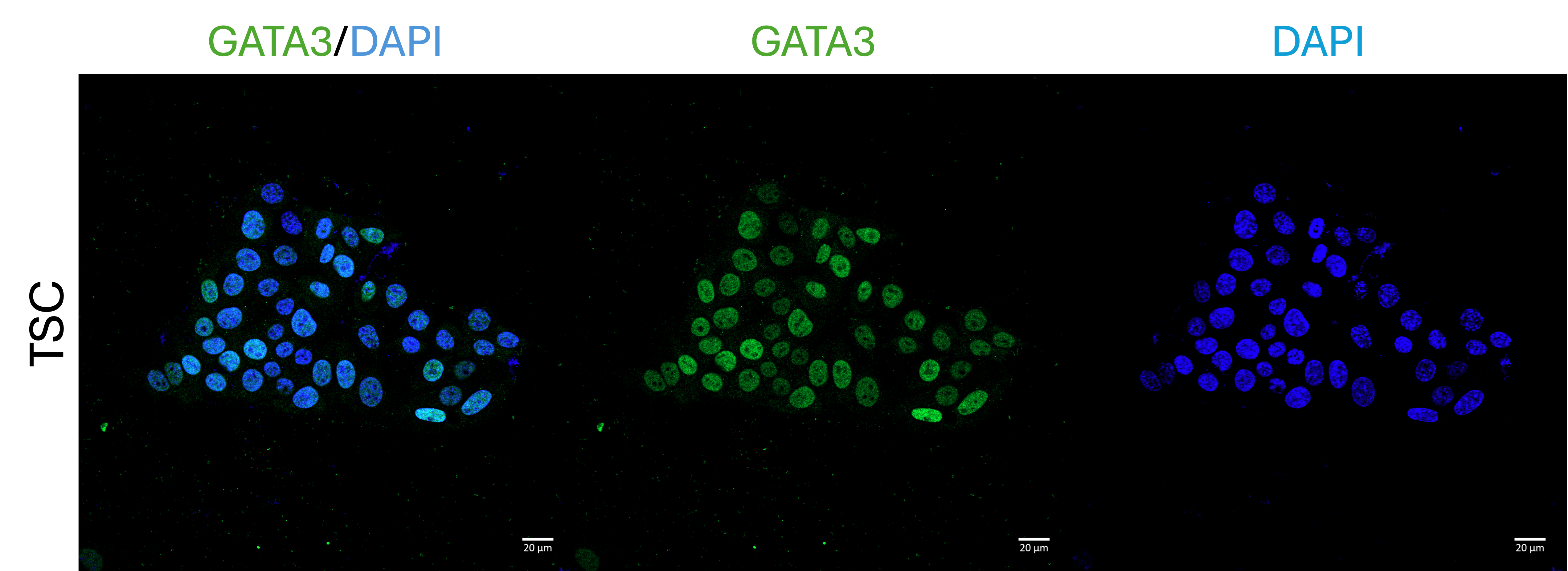

Supplement: Supplementary file 1 [file Image1.tif]
